# Barcoding Megadiversity: An Arthropod Database from Sites in the Neotropical Eastern Pacific Bioregion

**DOI:** 10.1038/s41597-026-07465-z

**Published:** 2026-06-03

**Authors:** Ana B. García-Ruilova, David A. Donoso, Yves Basset, Julio C. Carrión-Olmedo, Sandra Garcés-Carrera, Pablo Jarrín-V, Sofia I. Muñoz-Tobar, Sameer Padhye, Kate H. J. Perez, Jayme E. Sones, Diego Quiñónez-Sánchez, Pablo Sánchez, M. Alex Smith, Daniel H. Janzen, Winnie Hallwachs, Paul D. N. Hebert, Diego J. Inclán

**Affiliations:** 1 Instituto Nacional de Biodiversidad, Quito, Ecuador; 2 Departamento de Biología, Escuela Politécnica Nacional, Quito, Ecuador; 3 Smithsonian Tropical Research Institute, Panama City, Panama; 4 Smithsonian’s National Zoo and Conservation Biology Institute, Center for Species Survival, Front Royal, 22630 USA; 5 Instituto Nacional de Investigaciones Agropecuarias, Estación Experimental Santa Catalina, Quito, Ecuador; 6 Centre for Biodiversity Genomics, University of Guelph, Guelph, Ontario, Canada; 7 Department of Integrative Biology, University of Guelph, Guelph, ON N1G 2W1 Canada; 8 Guanacaste Dry Forest Conservation Fund, Área de Conservación Guanacaste, Apartado, 169-5000 Costa Rica; 9 Department of Biology, University of Pennsylvania, Philadelphia, PA 19104 USA; 10 Facultad de Ciencias Agrícolas, Universidad Central del Ecuador, Quito, Ecuador

## Abstract

We present the first dataset collection of arthropod diversity from ten terrestrial sites in the Neotropical Eastern Pacific Bioregion, in the territories of Costa Rica, Ecuador, Panama, and the Galapagos and Cocos Islands. The twenty–five datasets comprise 1–7 years of sampling. Together, we document a total of 952,087 arthropod specimens and 45,812 BINs. The datasets are distributed in 1 phylum, 7 classes, 39 orders, 556 families, and 2,318 genera. In the first year of sampling, the datasets revealed unprecedented arthropod diversity; notably, at Mashpi in Ecuador (n = 7,848 BINs) and Guanacaste in Costa Rica (n = 7,790 BINs), both exhibited the highest number of unique BINs. Arthropod abundance was greatest at Quetzales (n = 130,193 records) and Baru (n = 111,044 records) in Costa Rica. The five most abundant orders were Diptera (n = 20,901 BINs), Hymenoptera (n = 8,964 BINs), Coleoptera (n = 4,272 BINs), Lepidoptera (n = 3,634 BINs), and Hemiptera (n = 2,166 BINs). The dataset collection provides a robust baseline for future arthropod biodiversity research.

## Background & Summary

The global challenge posed by biodiversity loss is acutely manifested in nations of exceptional biological richness^1^. Most of the diversity in animals is concentrated among terrestrial arthropods, although the global number of species remains uncertain^2–4^. To date, over one million insect species have been formally described; yet, recent estimates suggest a total closer to 5.5 million species^5–7^. Efforts to document this large biodiversity are crucially needed^8,9^. The Neotropical Eastern Pacific Bioregion, widely referenced in marine biodiversity research, is increasingly invoked to describe the distribution patterns of terrestrial taxa^10,11^. This bioregion extends along the Neotropical corridor, from the tropical forests of northern Costa Rica to southern Ecuador, encompassing the Pacific coastal lowlands as well as the adjacent Andean foothills^10^. This bioregion, though covering only 0.25% of the world’s land area, harbors a disproportionately high concentration of global biodiversity^12–14^, including globally significant hotspots such as: the Tropical Andes, Oceanic Volcanic Archipelagos, and the Chocó–Darién ecoregion^15^. While its vertebrate and plant diversity is relatively well-studied^16,17^, its arthropod fauna, which performs critical ecosystem functions, remains profoundly under-documented^14^.

Efforts to approximate the number of tropical species include local-scale surveys. Basset *et al*.^18^ reported 6,144 insect species in half a hectare of the San Lorenzo Forest Reserve (Panama), a tropical forest in the Neotropical Eastern Pacific Bioregion. This suggests that a single hectare can harbor up to 64% of the 25,000 arthropod species expected across a 6,000-hectare reserve, with most of this arthropod diversity still undescribed. This profound knowledge gap is a major impediment to valuing biodiversity as a strategic resource, understanding ecological processes, assessing ecosystem health, and formulating effective conservation strategies. The urgency to document biodiversity is amplified by severe and ongoing threats to tropical ecosystems, particularly high rates of deforestation and global warming, which threaten to extinguish yet to be discovered species^19,20^. Furthermore, terrestrial arthropods are experiencing substantial declines, leading to significant losses in insect biodiversity and critical ecological functions, including pollination and decomposition^21–23^.

To address the critical need for documenting arthropod biodiversity in the Neotropical Eastern Pacific Bioregion, we have systematized the sampling efforts of the Global Malaise Program in Ecuador, Panama and Costa Rica. The primary output of this effort is the datasets described herein, that contribute to establishing a digital, and queryable biodiversity reference for arthropods in the bioregion. This resource provides a robust baseline for any future arthropod biodiversity research and eDNA or DNA metabarcoding studies in the Neotropical region. Furthermore, the datasets strategically address regional sampling weaknesses, which suffer from taxonomic and geographic gaps. By generating a large, high-quality, and geographically focused collection of datasets, using a single, standardized workflow, our work contributes to the global reference biodiversity library for one of the most biodiverse regions on Earth.

## Methods

### Study sites and sampling periods

The Neotropical Eastern Pacific Bioregion forms part of the terrestrial Neotropical Region^10^. It extends along the tropical Pacific slope of Central America and northwestern South America. The ten sampled sites and the sampling period (dd/mm/yyyy) for the corresponding twenty-five datasets were: 1) Cerro Blanco Protected Forest (Ecuador), 06/01/2020–01/02/2021; 2) Mashpi Protected Forest (Ecuador) 03/01/2020–07/01/2021; 3) Pululahua Geobotanical Reserve (Ecuador) 05/01/2020–14/01/2021; 4) Paluguillo Reserve (Ecuador) 11/03/2021–01/04/2022; 5) A pooled dataset representing Floreana, Isabela, San Cristobal, and Santa Cruz Islands (Galapagos, Ecuador) 30/06/2021–09/06/2022; 6) Barro Colorado Island (Panama) 10/05/2014–02/05/2015; 7) Central Pacific Conservation Area Baru (Costa Rica) 20/10/2006–13/04/2021; 8) Cocos Island National Park (Costa Rica) 06/08/2019–16/07/2020; 9) Guanacaste Conservation Area (Costa Rica) 14/11/2013–30/08/2020; and 10) Los Quetzales National Park (Costa Rica) 30/08/2019–30/08/2020. Aspects related to the specific coordinates of each sampling site and other characteristics are presented as part of the description of the datasets.

### Specimen and data sources

Datasets were obtained from the measurements and analyses of the specimens collected at each of the ten localities, as part of the Global Malaise Program (GMP), and coordinated by the iBOL partners Instituto Nacional de Biodiversidad (Ecuador), Smithsonian Tropical Research Institute (Panama), and Instituto Nacional de Biodiversidad (Costa Rica). Initiated in 2012, the GMP aims to assess the spatial and temporal variability of arthropod biodiversity at a global scale through the deployment of Malaise traps across diverse habitats worldwide^24^. The specimens described in these datasets were collected according to the standardized Malaise trap sampling protocol detailed by deWaard in 2019^25^. The protocol was designed to capture a wide range of flying insects and ensure comparability across sites and sampling periods. Malaise traps at the forest edge were consistently deployed at each locality, covering a variety of altitudinal, climatic, and ecological gradients. The deployment of traps enabled the exploration of spatial patterns of diversity, taxonomic composition, and species turnover. To facilitate the interpretation of biodiversity data for the Galapagos, we treated samples from the four islands as a single pooled locality.

### Specimen processing, DNA barcode analysis, and Barcode Index Numbers (BINs)

At the Centre for Biodiversity Genomics (University of Guelph), specimens were arrayed using high-throughput automated systems^25,26^. DNA was extracted via a magnetic bead protocol, and the COI barcode region was sequenced using the PacBio Sequel SMRT platform^27^. Sequences were validated, uploaded to the Barcode of Life Data Systems (BOLD), and clustered into Barcode Index Numbers (BINs) using the RESL algorithm^28^ for species proxy determination. The BIN system is a provisional taxonomic framework that assigns unique identifiers to clusters of COI barcode sequences. Each BIN represents a molecular operational taxonomic unit (MOTU), based on sequence divergence^28^. Individual records are assigned either to an existing BIN or to a new BIN, but only sequences that meet quality criteria are included in the RESL analysis. These criteria include a standard barcode region coverage of 500 base pairs (bp), less than 1% ambiguous bases, and the absence of stop codons or evidence of sequence contamination. We used only those sequences that had been assigned to a BIN in our datasets. The RESL analysis allows sequence records containing more than 300 bp of the barcode region (between positions 70 and 700 of the BOLD alignment) to be included in existing BINs, whereas records establishing a new BIN must include more than 500 bp of the barcode region^28,29^. A taxonomic identification for a particular BIN was possible only if all specimens within that BIN shared the same taxonomic identification at the family, genus, or species level. In cases of taxonomic discordance among specimens within a BIN, the identification was assigned at the less inclusive taxonomic level above the point of conflict. Taxonomic identification was based on DNA barcodes and morphology^30–35^.

### Dataset systematization

To ensure data quality, all records were curated through a combination of manual verification and computational filtering, retaining only those specimens with an assigned Barcode Index Number (BIN). Records were excluded if they exhibited any of the following issues: (1) evidence of contamination, (2) the presence of stop codons, or (3) a flagged status within the BOLD system. The validated records were then integrated into ten datasets. These datasets are organized by sampling locality and include georeferenced collection data, hierarchical taxonomic identification, the corresponding DNA barcode sequence, and the associated BIN designation.

### Biodiversity patterns

To describe the salient features and demonstrate the utility of these datasets for arthropod biodiversity research, we present a statistical overview of the data. All localities have a single year of sampling, except for localities Baru and Guanacaste, with 1.8 years and 6.8 years correspondingly. Thus, to provide an accurate overview of biodiversity in these datasets we prepared “Data Overview” section based on all available years per sampling site, followed by a focused description of the first year of sampling across localities. The datasets were compared according to their corresponding first year of sampling. Data processing was conducted in R^36^, utilizing specialized packages for sequence retrieval, manipulation, and ecological analyses. The BOLDconnectR package^37^ provided direct access to the Barcode of Life Data System (BOLD) data. Additional packages: dplyr^38^, tidyr^39^, ggplot2^40^, ggrepl^41^, viridis^42^, tibble^43,44^, lubridate^45^, patchwork^46^, forcats^38^, UpSetR^47^, iNEXT^48,49^, sf^50^, tidyverse^51^, and scales^52^ facilitated data wrangling, visualization, and dataset comparisons. All data manipulation and visualization scripts are described in Carrion-Olmedo^53^. Datasets were imported in R for each locality using the bold.fetch() function.

Species accumulation and diversity patterns were evaluated using the Hill number framework, focusing on q = 0, which corresponds to species richness. Abundance data were organized into locality-specific vectors and analyzed with the iNEXT package^48,49^ to generate rarefaction and extrapolation curves. iNEXT estimates diversity under standardized sampling conditions by interpolating and extrapolating species richness as a function of both sample size and sample coverage, allowing direct comparisons among sites with uneven sampling effort. For each locality, we computed rarefied and extrapolated richness up to twice the reference sample size and obtained confidence intervals following the bootstrap procedure implemented in iNEXT.

Two complementary graphical outputs were generated for q = 0: (i) sample-size–based rarefaction/extrapolation curves, which illustrate how BIN richness increases with additional sampling effort, and (ii) coverage-based curves, which standardize comparisons by sample completeness rather than sampling intensity.

We assessed the degree to which localities shared the Barcode Index Numbers (BINs). An intersection matrix, based on the presence or absence of BINs per locality, was processed with the package UpSetR^47^ to visualize shared BINs. Localities were represented as points sized by BIN richness and connected by lines proportional to the number of exclusive shared BINs, with counts annotated at line midpoints.

## Data Records

**Fig. 1:**
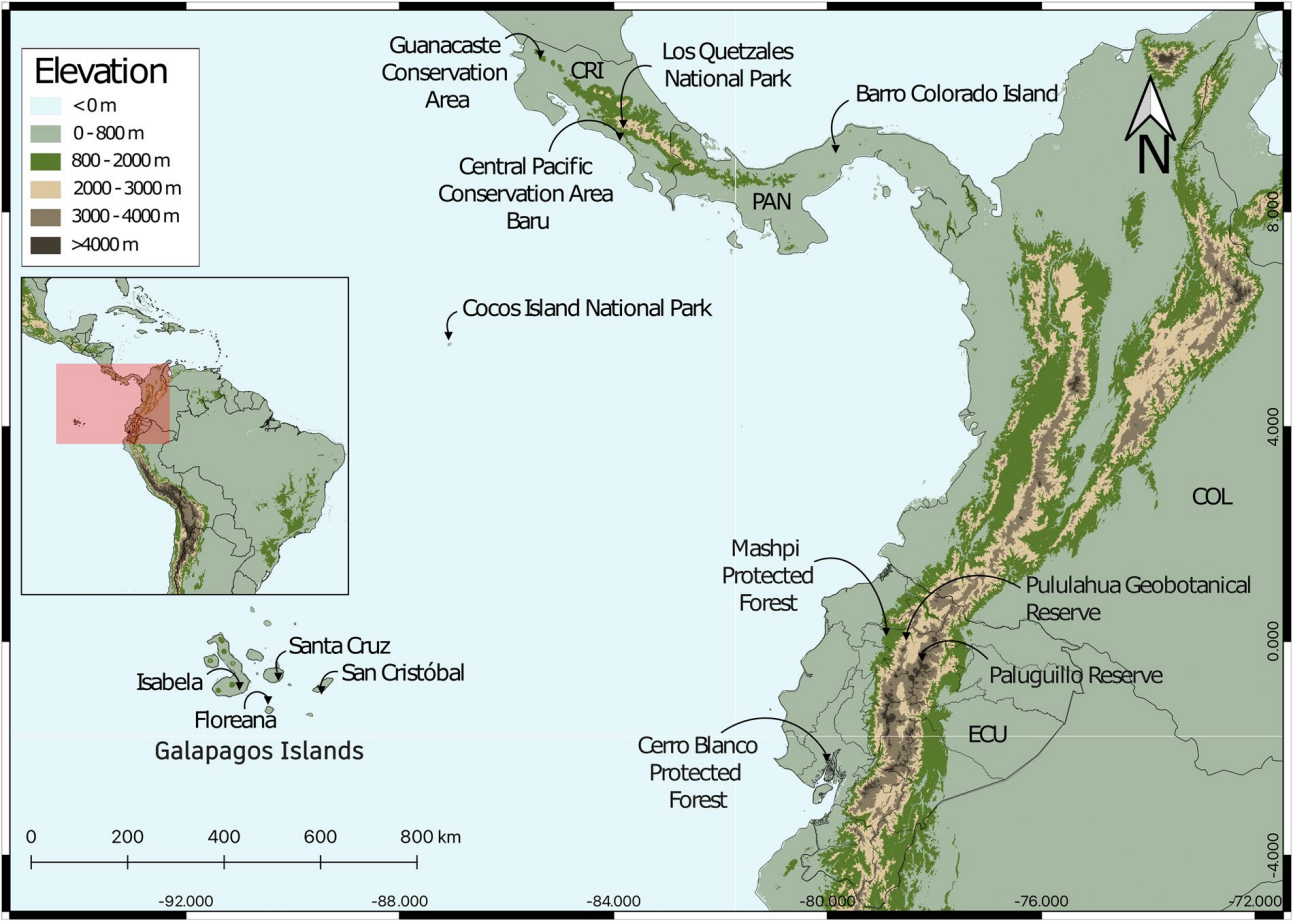
Geographic distribution of collection sites across western Ecuador, Galapagos, Cocos Island, Panama, and Costa Rica for the 20 presented datasets.

All arthropod diversity data in the Neotropical Eastern Pacific Bioregion are stored in twenty-five datasets and available for direct download from the FIGSHARE data repository^54^. The datasets describe a total of 952,087 arthropod specimens and 45,812 BINs in a range of elevations between 5 and 3,767 meters across different localities (Fig. 1).

The datasets are organized into twenty-five folders according to sampling localities listed in Supplementary Table S1. Each folder contains three files JSON, TSV (Tab-Separated Values), or DwC (Darwin Core) formats to facilitate interoperability and data analysis. The files collectively include 66 data fields, consistent with those provided in the Combined Data download. These fields are grouped into several categories describing taxonomic, specimen, geographic, sequence, and analytical information.

Taxonomic information includes more than ten fields spanning hierarchical ranks from Kingdom to Species, together with associated identifiers. Specimen-level metadata includes voucher ID, holding institution, collection date, and collector name. Geographic information includes country, province, specific locality, geographic coordinates, and depth or elevation when available. Sequence-related data includes the marker code, GenBank accession number, and the corresponding DNA sequence. Analytical fields include the Barcode Index Number (BIN) URL and the identification method used. These tabular formats contain several attributes that are described in Supplementary Table S2.

## Data Overview

### Diversity and taxonomic composition of the datasets

**Fig. 2:**
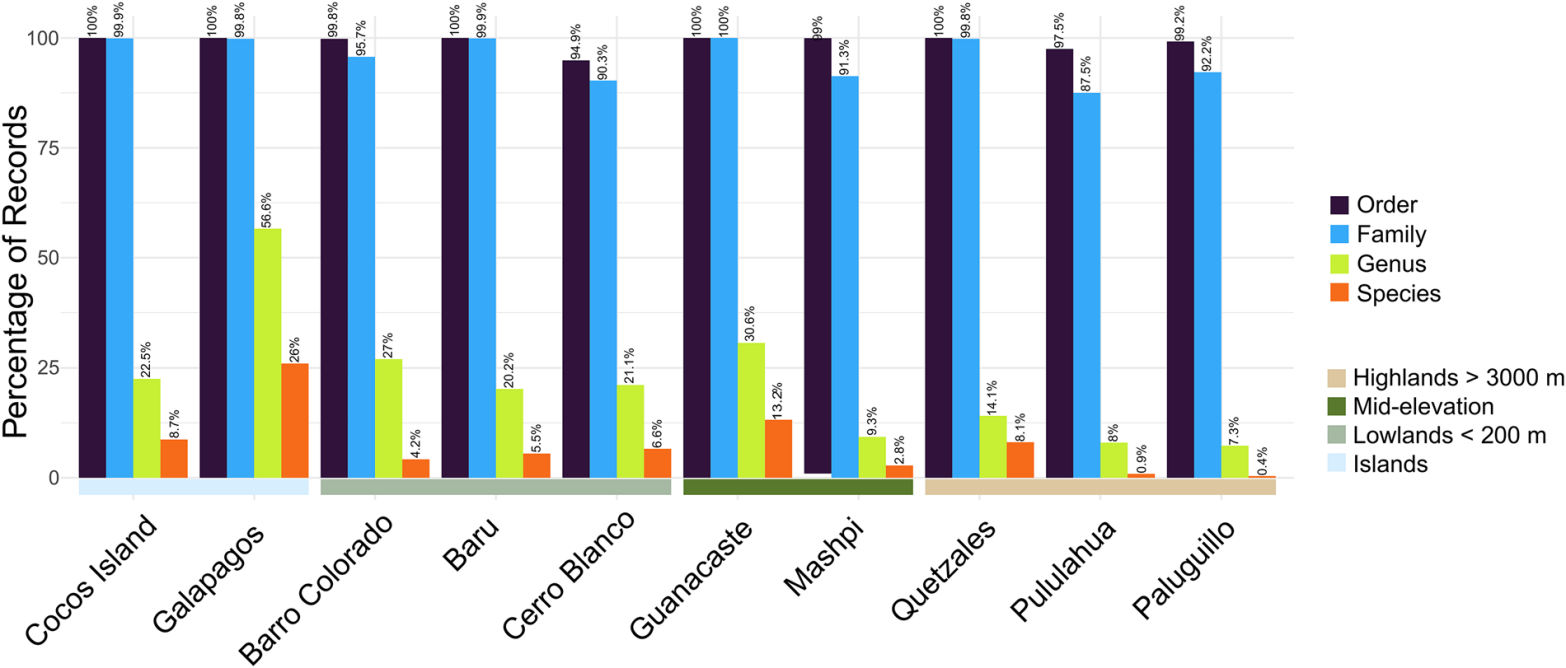
Taxonomic resolution of records among the 10 sampling sites. Bar height corresponds to the percentage of specimens assigned to Order, Family, Genus or Species level. Sampling sites were sorted across increasing elevation from left to right.

The presented datasets for the 10 sampled localities comprise 1 phylum, 7 classes, 39 orders, 556 families, and 2,318 genera of arthropods. Across all localities and the available extent of sampling time periods, taxonomic resolutions were achieved predominantly at the order and family levels. Only a small fraction of specimens could be resolved to genus, and an even smaller proportion to species. The datasets from the Galapagos Islands and the mid-elevation sites in Guanacaste exhibited the highest taxonomic resolution, with 56% and 37% of records identified to genus-level, and 26% and 20% identified to species-level, respectively (Fig. 2).

**Fig. 3:**
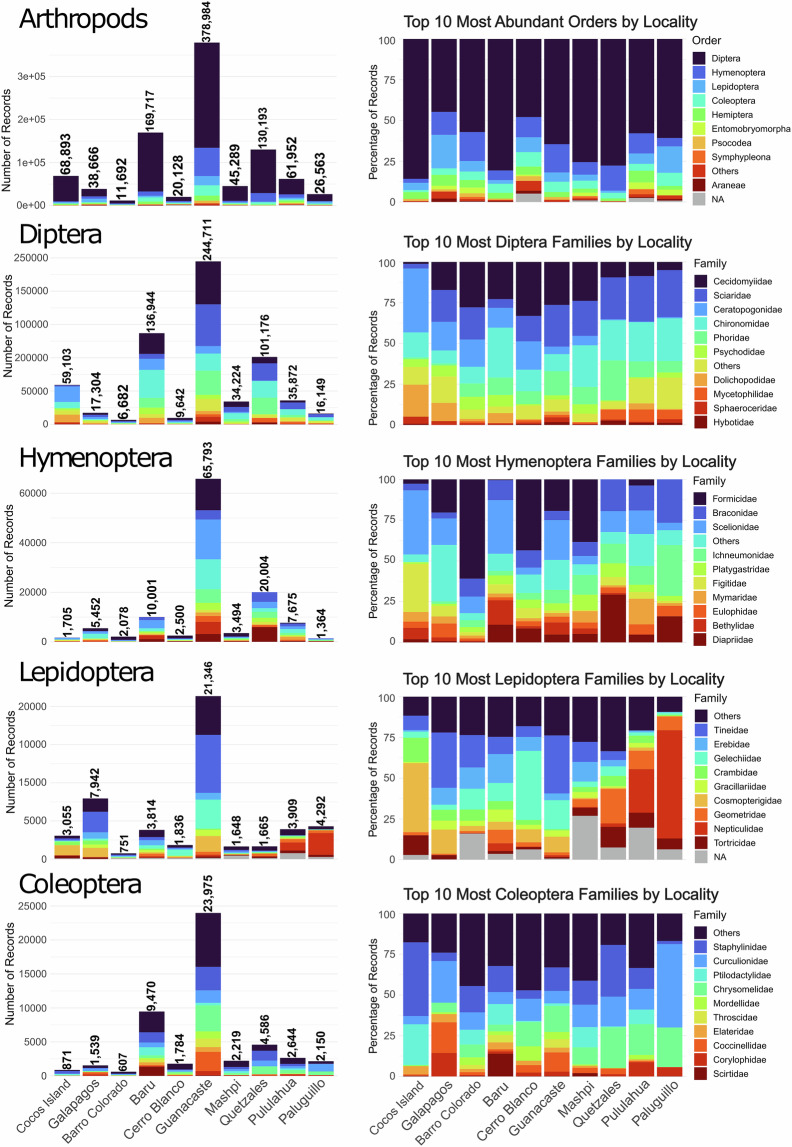
Taxonomic composition of Malaise traps across sampling sites and the full available sampling time. Raw (left panels) and relative (right panels) abundance of taxa in the 10 sampling sites sorted across increasing elevation from left to right.

The taxonomic composition of Malaise trap samples reveals a consistent dominance of the order Diptera across all 10 sampled sites, regardless of elevation or raw abundance (Fig. 3). In terms of raw specimen counts, Guanacaste stands out as the site with the most data and longer sampling times, recording significantly higher numbers of records for Diptera, Hymenoptera, Lepidoptera, and Coleoptera compared to other localities. While Diptera remains the most specimen-rich group, the relative abundance of other orders shifts across the elevational gradient; for instance, Hymenoptera represents a more substantial percentage of records in island sites like Cocos and Galapagos Islands compared to several mainland highland sites. At the family level within Diptera, Cecidomyiidae and Sciaridae are consistently prevalent, though their relative proportions vary, with Sciaridae showing increased dominance in high-elevation sites like Paluguillo.

**Fig. 4:**
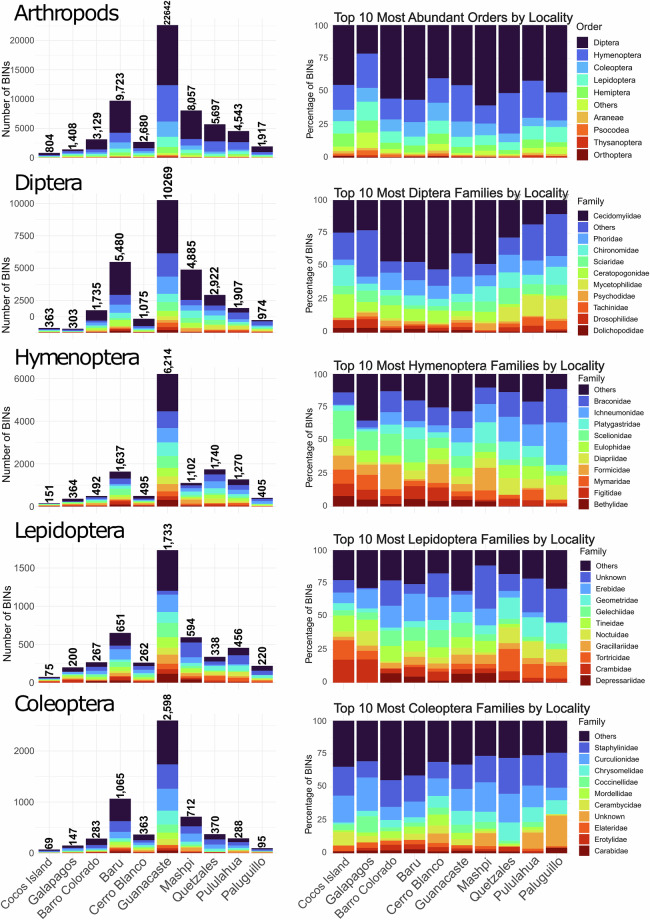
BINs composition of Malaise traps across sampling sites and the full available sampling time. Raw (left panels) and relative (right panels) abundance of BINs in the 10 sampling sites sorted across increasing elevation from left to right.

The composition of Barcode Index Numbers (BINs) mirrors these broad taxonomic trends but provides a more nuanced view of biodiversity (Fig. 4). Similar to raw specimen counts, the highest BIN richness is concentrated in Guanacaste, followed by Baru, reflecting the longer sampling times at both sites. Interestingly, while Diptera also dominates the BIN counts, the relative diversity of Hymenoptera BINs is notably high across all sites, often exceeding its relative specimen abundance. Within specific orders, such as Lepidoptera, the BIN distribution highlights a high turnover of families across localities, with “Others” and “Unknown” categories making up a significant portion of the genetic diversity, particularly in sites like Mashpi and Pululahua.

**Fig. 5:**
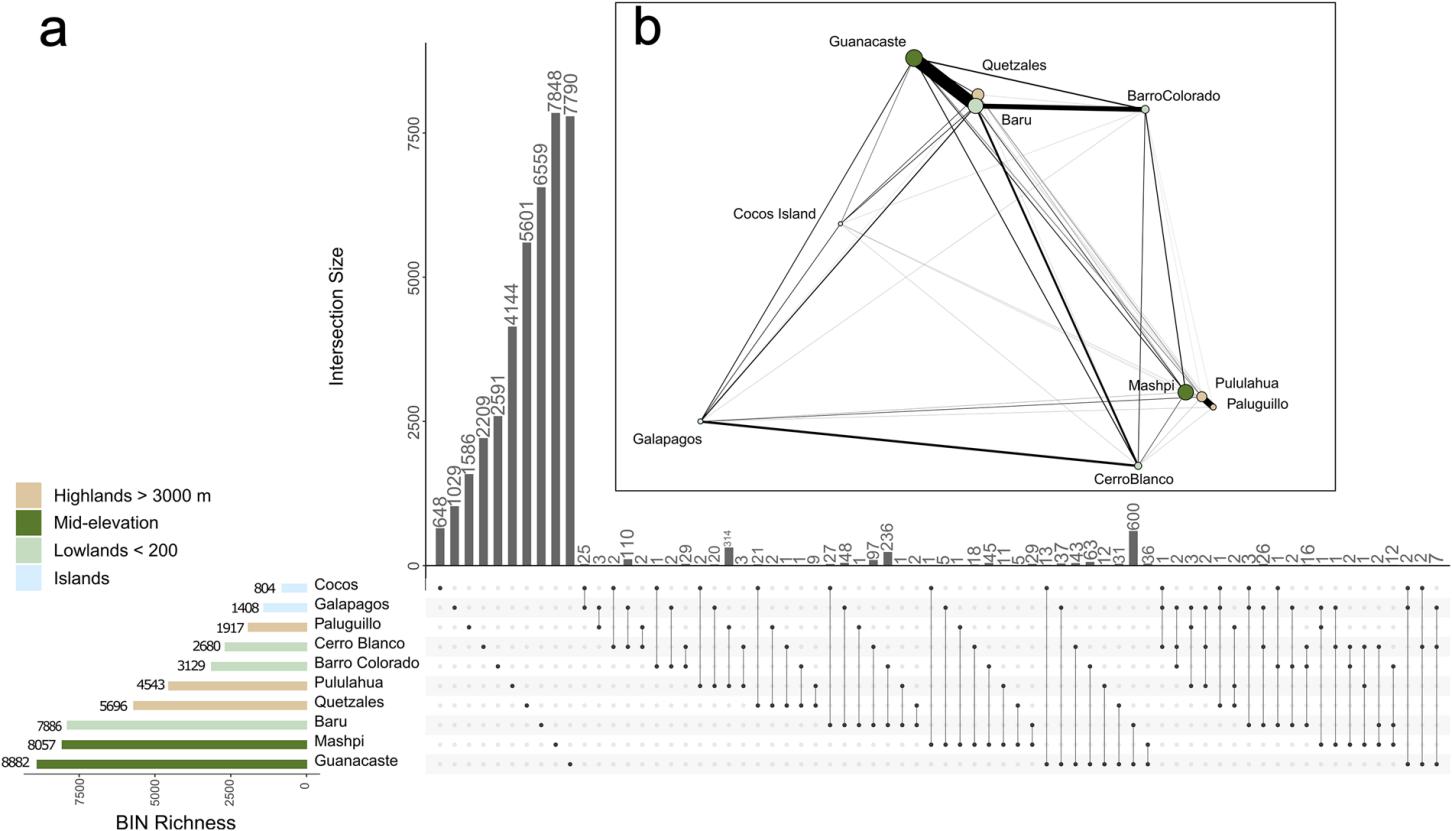
Distribution and overlap of Barcode Index Numbers (BINs) across ten sampling sites for the first year of sampling. a) An **´**UpSet´ plot of the sampling sites illustrating the number of unique and shared BINs sets. The connected dot pairs illustrate shared groups between sampling sites, quantified by the corresponding bar height and numbers above connections. Single dots and their corresponding bars correspond to unique BIN counts. b) An inset network showing euclidean distances between study sites and relative abundance. The width of the connecting lines are the proportion of shared BINs between sites and the size of the dots represent BIN richness.

Considering only those localities with a single year of sampling, Mashpi excels as the one with the highest number of recorded BINs (n = 8,057). Diptera is the most diverse order in Mashpi, whereas Hymenoptera, Lepidoptera, and Coleoptera show greater BIN richness in Guanacaste, consistent with its elevational range (800–2,000 m a.s.l.). The lowest BIN richness values were recorded for Cocos Island and Galapagos. For one-year sampled localities, Mashpi stands out with the highest number of unique BINs (n = 7,848 unique BINs), an indicator of notable endemism (Fig. 5).The locality with the second largest number of unique BINs was Guanacaste (n = 7,790), and the third was Baru (n = 6,559); in contrast to Mashpi, Guanacaste and Baru have been sampled for over one year (Fig. 5). Within the first year of sampling, the strongest taxonomic sharing occurs between geographically or ecologically linked sites, such as the prominent connection between Guanacaste and Baru, or the clustering of highland sites like Pululahua and Paluguillo (Fig. 5).

The analysis conveyed by the “UpSet” plot (Fig. 5) highlights that while a vast majority of BINs are unique to specific localities, there is a complex network of shared taxa between sites, though these intersections represent a smaller fraction of the total diversity compared to site-specific richness. Mid-elevation and highland mainland sites dominate overall richness compared to the more isolated island and lowland localities. Together, these results suggest that while regional biodiversity is immense, it is characterized by high turnover and localized endemism across different elevations and geographic barriers.

**Fig. 6:**
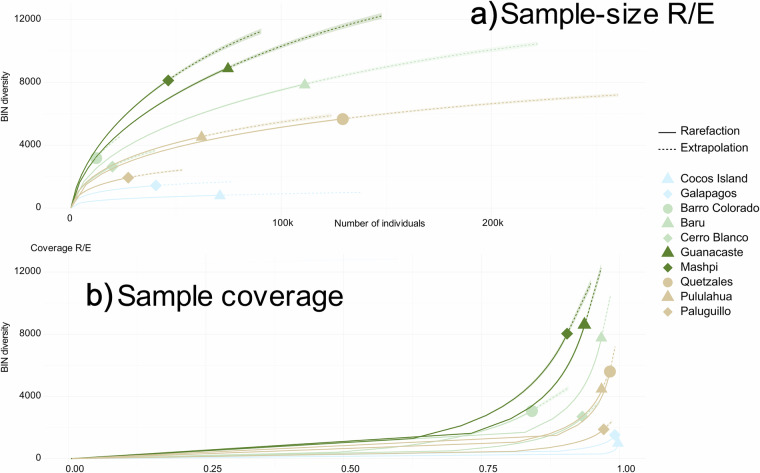
Rarefaction and extrapolation curves to assess Barcode Index Number (BIN) diversity and sampling completeness across ten distinct localities. Top: Sample-size–based curves illustrate the expected increase in observed richness as a function of additional sequencing depth, highlighting differences in sampling effort and richness accumulation among localities. Bottom: Coverage-based curves standardize diversity estimates by sample completeness, enabling comparisons across sites despite unequal sampling intensities.

The sample-size-based rarefaction and extrapolation curves demonstrate significant variation in taxonomic richness, with Mashpi and Guanacaste exhibiting the highest rates of accumulation and overall BIN diversity, while island sites such as Cocos Island and Galapagos show the lowest (Fig. 6, top panel). The steep, non-asymptotic trajectories for most mainland sites, particularly Baru and Cerro Blanco, suggest that despite high sequencing depth (exceeding 100k–200k individuals in some cases), a substantial portion of the biodiversity remains unsampled (Fig. 6, top panel). While some sites like Cocos Island approach a sample coverage of 1.0 (indicating high completeness), the hyper-diverse sites like Mashpi and Guanacaste still possess steep curves even at high coverage levels, signifying a vast “long tail” of rare BINs (Fig. 6, bottom panel). This contrast emphasizes that the observed differences in richness are not artifacts of sampling effort; rather, these reflect ecological variations in community structure and species evenness across the 10 sampled sites.

## Technical Validation

To validate DNA sequence records for each dataset, we employed the Barcode Index Number (BIN) discordance analysis, based on the Refined Single Linkage Algorithm (RESL) in BOLD^29^ and the Taxon-ID Tree, constructed using the Neighbor Joining (NJ) method and Kimura 2-Parameter (K2P) distances. After applying these algorithms, all records were assigned to an existing BIN, or if divergence from existing BINs was greater than 2.2%, a new BIN was created for assignment. Records were included in the RESL analysis only if they met specific quality criteria, including a standard barcode region coverage of 500 base pairs (bp) for the assignment of new BINs, and sequences longer than 300 bp for assignment to existing BINs, less than 1% of ambiguous bases, and the absence of stop codons or evidence of sequence contamination. Additionally, each biological specimen described in the datasets underwent verification through photographic documentation and validation of georeferenced information to ensure data reliability and traceability. The results of the taxonomic resolution analysis indicate a strong overall congruence between traditional taxonomy and COI sequence data. Nonetheless, certain taxa and species groups remain insufficiently studied and further taxonomic and molecular investigations are required to clarify unresolved or complex cases.

## Usage Notes

The FIGSHARE datasets are a resource for assessing species diversity in the Neotropical Eastern Pacific Bioregion. These serve as a valuable reference library for future studies involving DNA barcoding, metabarcoding, and environmental DNA (eDNA) monitoring, providing a solid foundation for the molecular identification of arthropods and other associated taxa. The datasets provide a reference tool for researchers, environmental managers, and policymakers seeking to understand regional biodiversity patterns, endemism levels, and temporal dynamics of the sampled communities. The datasets represent a portion of the broader Global Malaise Program, a long-term initiative aimed at documenting arthropod diversity at a global scale. Thus, the datasets presented herein not only synthesize the current state of knowledge for the Neotropical region but also open new avenues of research into the ecological and evolutionary processes that shape tropical biodiversity. In addition to FIGSHARE^54^ the datasets are also hosted at the Barcode of Life Data Systems (BOLD)^55–79^ under their corresponding DOIs (see Supplementary Table S1), and can also be downloaded directly via the BOLD API (www.boldsystems.org/index.php/resources/api) or by using the BOLDconnectR package in R, thereby facilitating their integration into automatized bioinformatic and ecological analyses. A description of the outline of the twenty-five datasets at the BOLD repository is available in Supplementary information 1. Because of storage space limitations, the images of the specimens in the datasets are solely available in BOLD^55–79^. Supplementary Table S1 has the corresponding hyperlinks to all datasets for accessing to the specimen images.

## Supplementary information

Supplementary Information

## Data Availability

Users can access the primary data records via the following Digital Object Identifier (DOI) in FIGSHARE: 10.6084/m9.figshare.31633891^54^.

## Code availability

The scripts and workflows used for the biodiversity analyses were made available by Carrion-Olmedo^53^ at https://github.com/juliocesarcaol/Metacommunities_Malaise.
