# Supplementary material for: Barcoding Megadiversity: An Arthropod Database from Sites in the Neotropical Eastern Pacific Bioregion
Source: Sci Data. 2026 Jun 3;13:1026. doi: 10.1038/s41597-026-07465-z (PMC13356025; doi:10.1038/s41597-026-07465-z)
Supplement: Supplementary file 1 — Supplementary Information [file 41597_2026_7465_MOESM1_ESM.docx]

**Supplementary Material – Tables and additional information in the BOLD repository**

**Title:** Barcoding Megadiversity: An Arthropod Database from Sites in the Neotropical Eastern Pacific Bioregion

**Authors:** Ana B. García-Ruilova, David A. Donoso, Yves Basset, Julio C. Carrión-Olmedo, Sandra Garcés-Carrera, Pablo Jarrín-V., Sofia I. Muñoz-Tobar, Sameer Padhye, Kate H. J. Perez, Jayme E. Sones, Diego Quiñónez-Sánchez, Pablo Sánchez, M. Alex Smith, Daniel H. Janzen, Winnie Hallwachs, Paul D. N. Hebert, Diego J. Inclán.

Correspondence to:

Ana B. García Ruilova: [abgarciar18@gmail.com](mailto:abgarciar18@gmail.com)

**Table S1. Provenance location, repository identifiers, and additional dataset metadata**

| **Locality of provenance** | **Folder title in Figshare** | **Dataset Code in BOLD** | **Dataset DOI in BOLD** | **Coordinates long; lat (decimal degrees)** | **Elevation (meters above sea level)** | **Country (three-letter code)** | **Specimens** | **BINs** | **Unique BINs** |
| --- | --- | --- | --- | --- | --- | --- | --- | --- | --- |
| Cocos Island National Park | DS-CRMCO3_GMP - Cocos Edge Trap 2019-2021. Part 1 | DS-CRMCO3 | [dx.doi.org/10.5883/DS-CRMCO3](http://dx.doi.org/10.5883/DS-CRMCO3) | -87,083; 5.533 | 60 | CRI | 45,639 | 715 | 85 |
| Cocos Island National Park | DS-CRMCO4_GMP - Cocos Edge Trap 2019-2021. Part 2 | DS-CRMCO4 | [dx.doi.org/10.5883/DS-CRMCO4](http://dx.doi.org/10.5883/DS-CRMCO4) | -87,083; 5.533 | 60 | CRI | 23,251 | 512 | 29 |
| Central Pacific Conservation Area Baru | DS-CRMBA1_GMP - Baru Edge Trap 2019-2021. Part 1 | DS-CRMBA1 | [dx.doi.org/10.5883/DS-CRMBA1](http://dx.doi.org/10.5883/DS-CRMBA1) | -83.874; 9.262 | 15 | CRI | 44,432 | 5,166 | 434 |
| Central Pacific Conservation Area Baru | DS-CRMBA2_GMP - Baru Edge Trap 2019-2021. Part 2 | DS-CRMBA2 | [dx.doi.org/10.5883/DS-CRMBA2](http://dx.doi.org/10.5883/DS-CRMBA2) | -83.874; 9.262 | 15 | CRI | 19,328 | 3,295 | 223 |
| Central Pacific Conservation Area Baru | DS-CRMBA3_GMP - Baru Edge Trap 2019-2021. Part 3 | DS-CRMBA3 | [dx.doi.org/10.5883/DS-CRMBA3](http://dx.doi.org/10.5883/DS-CRMBA3) | -83.874; 9.262 | 15 | CRI | 47,270 | 4,513 | 364 |
| Central Pacific Conservation Area Baru | DS-CRMBA4_GMP - Baru Edge Trap 2019-2021. Part 4 | DS-CRMBA4 | [dx.doi.org/10.5883/DS-CRMBA4](http://dx.doi.org/10.5883/DS-CRMBA4) | -83.874; 9.262 | 15 | CRI | 46,357 | 4,588 | 379 |
| Central Pacific Conservation Area Baru | DS-CRMBA5_GMP - Baru Edge Trap 2019-2021. Part 5 | DS-CRMBA5 | [dx.doi.org/10.5883/DS-CRMBA5](http://dx.doi.org/10.5883/DS-CRMBA5) | -83.874; 9.262 | 15 | CRI | 12,313 | 2,331 | 149 |
| Cerro Blanco Protected Forest | DS-EMCEB_ GMP - Cerro Blanco Edge Trap 2020-2021 | DS-EMCEB | [dx.doi.org/10.5883/DS-EMCEB](http://dx.doi.org/10.5883/DS-EMCEB) | -80.015; -2.182 | 38 | ECU | 20,128 | 2,656 | 1,864 |
| Galapagos - Floreana Island | DS-EMFLO_ GMP - Floreana Edge Trap 2021-2022 | DS-EMFLO | [dx.doi.org/10.5883/DS-EMFLO](http://dx.doi.org/10.5883/DS-EMFLO) | -90,443; -1,312 | 360 | ECU | 7,803 | 431 | 85 |
| Galapagos - Santa Cruz Island | DS-EMSCZ_ GMP - Santa Cruz Edge Trap 2021-2022 | DS-EMSCZ | [dx.doi.org/10.5883/DS-EMSCZ](http://dx.doi.org/10.5883/DS-EMSCZ) | -90,269; -0.668 | 130 | ECU | 13,268 | 768 | 106 |
| Galapagos - San Cristóbal Island | DS-EMSCB_ GMP - San Cristóbal Edge Trap 2021-2022 | DS-EMSCB | [dx.doi.org/10.5883/DS-EMSCB](http://dx.doi.org/10.5883/DS-EMSCB) | -89,462; -0.921; | 166 | ECU | 7,767 | 650 | 135 |
| Galapagos - Isabela Island | DS-EMISA_GMP - Isabela Edge Trap 2021-2022 | DS-EMISA | [dx.doi.org/10.5883/DS-EMISA](http://dx.doi.org/10.5883/DS-EMISA) | -90,997; -0.845 | 169 | ECU | 9,828 | 640 | 96 |
| Barro Colorado Island | DS-MALPTY_ Malaise trap samples, Barro Colorado Island, Panama-2014 | DS-MALPTY | [dx.doi.org/10.5883/DS-MALPTY](http://dx.doi.org/10.5883/DS-MALPTY) | -79.847; 9.151 | 162 | PAN | 12,722 | 3,130 | 1,621 |
| Guanacaste Conservation Area | DS-CRMGU1_GMP - Guanacaste Edge Trap 2014-2021. Part 1 | DS-CRMGU1 | [dx.doi.org/10.5883/DS-CRMGU1](http://dx.doi.org/10.5883/DS-CRMGU1) | -85.334; 10.763 | 820 | CRI | 62,528 | 8,096 | 274 |
| Guanacaste Conservation Area | DS-CRMGU2_GMP - Guanacaste Edge Trap 2014-2021. Part 2 | DS-CRMGU2 | [dx.doi.org/10.5883/DS-CRMGU2](http://dx.doi.org/10.5883/DS-CRMGU2) | -85.334; 10.763 | 820 | CRI | 82,010 | 7,615 | 224 |
| Guanacaste Conservation Area | DS-CRMGU3_GMP - Guanacaste Edge Trap 2014-2021. Part 3 | DS-CRMGU3 | [dx.doi.org/10.5883/DS-CRMGU3](http://dx.doi.org/10.5883/DS-CRMGU3) | -85.334; 10.763 | 820 | CRI | 63,881 | 8052 | 261 |
| Guanacaste Conservation Area | DS-CRMGU4_GMP - Guanacaste Edge Trap 2014-2021. Part 4 | DS-CRMGU4 | [dx.doi.org/10.5883/DS-CRMGU4](http://dx.doi.org/10.5883/DS-CRMGU4) | -85.334; 10.763 | 820 | CRI | 67,014 | 6,611 | 214 |
| Guanacaste Conservation Area | DS-CRMGU5_GMP - Guanacaste Edge Trap 2014-2021. Part 5 | DS-CRMGU5 | [dx.doi.org/10.5883/DS-CRMGU5](http://dx.doi.org/10.5883/DS-CRMGU5) | -85.334; 10.763 | 820 | CRI | 62,371 | 8,631 | 484 |
| Guanacaste Conservation Area | DS-CRMGU6_GMP - Guanacaste Edge Trap 2014-2021. Part 6 | DS-CRMGU6 | [dx.doi.org/10.5883/DS-CRMGU6](http://dx.doi.org/10.5883/DS-CRMGU6) | -85.334; 10.763 | 820 | CRI | 41041 | 6,718 | 206 |
| Mashpi Protected Forest | DS-EMMAS_GMP - Mashpi Edge Trap 2020-2021 | DS-EMMAS | [dx.doi.org/10.5883/DS-EMMAS](http://dx.doi.org/10.5883/DS-EMMAS) | -78.884; 0.166 | 933 | ECU | 45,270 | 8,028 | 6,936 |
| Los Quetzales National Park | DS-CRMQU1_GMP - Quetzales Edge Trap 2019-2021. Part 1 | DS-CRMQU1 | [dx.doi.org/10.5883/DS-CRMQU1](http://dx.doi.org/10.5883/DS-CRMQU1) | -83.819; 9.614 | 3000 | CRI | 41,605 | 3,187 | 395 |
| Los Quetzales National Park | DS-CRMQU2_GMP - Quetzales Edge Trap 2019-2021. Part 2 | DS-CRMQU2 | [dx.doi.org/10.5883/DS-CRMQU2](http://dx.doi.org/10.5883/DS-CRMQU2) | -83.819; 9.614 | 3000 | CRI | 36,917 | 2,995 | 364 |
| Los Quetzales National Park | DS-CRMQU3_GMP - Quetzales Edge Trap 2019-2021. Part 3 | DS-CRMQU3 | [dx.doi.org/10.5883/DS-CRMQU3](http://dx.doi.org/10.5883/DS-CRMQU3) | -83.819; 9.614 | 3000 | CRI | 51,664 | 3979 | 776 |
| Pululagua Geobotanical Reserve | DS-EMPUL_ GMP - Pululahua Edge Trap 2020-2021 | DS-EMPUL | [dx.doi.org/10.5883/DS-EMPUL](http://dx.doi.org/10.5883/DS-EMPUL) | -78.502; 0.022 | 3003 | ECU | 61,919 | 4,534 | 3,351 |
| Paluguillo Reserve | DS-EMPAL_ GMP - Paluguillo Edge Trap 2021-2022 | DS-EMPAL | [dx.doi.org/10.5883/DS-EMPAL](http://dx.doi.org/10.5883/DS-EMPAL) | -78.231; -0.306 | 3767 | ECU | 26,563 | 1,914 | 1,435 |

**Table S2. Dataset attributes and their equivalents in the Darwin Core (DwC) terms**

| **Specimen data** | **Darwin Core equivalent (DwC)** | **Description** | **Format / Unit** |
| --- | --- | --- | --- |
| **Process ID** | materialSampleID / catalogNumber | Unique identifier assigned to each specimen or sample in BOLD. | Text (e.g., “BIOUG12345-A01”). |
| **Sample ID / Specimen ID** | materialSampleID | Internal code of the specimen within the project or institution. | Text. |
| **Institution Storing / Collection Code** | institutionCode, collectionCode | Institution or collection holding the physical specimen. | Text (e.g., “INABIO”, “BIOUG”). |
| **Sample Status** | (no direct DwC equivalent) / occurrenceStatus | Record status (e.g., “public”, “private”, “processed”). | Text. |
| **Identification ID** | occurrenceID / identificationID | Unique identifier of the taxonomic identification. | Text or UUID. |
| **Taxonomy (Full classification)** | kingdom, phylum, class, order, family, genus, species | Complete taxonomic hierarchy of the specimen. | Text (scientific name and authority). |
| **BIN (Barcode Index Number)** | taxonConceptID | Molecular cluster automatically assigned by the RESL algorithm (species-level proxy). | BIN code (e.g., “BOLD:AAB1234”). |
| **BIN URI / BIN Page** | references / associatedSequences | URL of the BIN public page in BOLD. | URL. |
| **Identification Method** | identificationRemarks / identificationQualifier | Method used for identification (morphological, COI, integrative, etc.). | Text. |
| **Identifier / Determined by** | identifiedBy | Name(s) of the person(s) who identified the specimen. | Text. |
| **Collection Date** | eventDate | Date of specimen collection. | ISO 8601 (YYYY-MM-DD). |
| **Collectors** | recordedBy | Name of collector(s) or collecting team. | Text. |
| **Collection Site / Locality** | locality | Description of the collection site. | Free text. |
| **Country** | country | Country where the specimen was collected. | Text (ISO 3166 English standard). |
| **Province / State** | stateProvince | Administrative division such as province, state, or department. | Text. |
| **Region / Municipality** | county / municipality | Lower-level administrative area or locality. | Text. |
| **Latitude** | decimalLatitude | Geographic latitude of the collection site. | Decimal degrees (e.g., −0.0567). |
| **Longitude** | decimalLongitude | Geographic longitude of the collection site. | Decimal degrees (e.g., −78.6873). |
| **Coordinate Precision** | coordinateUncertaintyInMeters | Estimated uncertainty of the coordinate measurement. | Numeric value (meters). |
| **Elevation** | minimumElevationInMeters / maximumElevationInMeters | Recorded elevation or altitudinal range. | Meters above sea level (m a.s.l.). |
| **Habitat / Site Description** | habitat | Description of the habitat or environmental setting (e.g., forest, mangrove, grassland). | Free text. |
| **Collection Code / Event ID** | eventID / samplingProtocol | Event code or field sampling protocol reference. | Text. |
| **Tissue Type** | preparations | Type of tissue used for molecular analysis. | Text (e.g., “leg”, “thorax”, “abdomen”). |
| **Extraction ID** | associatedSequences | Identifier of the DNA extract used for sequencing. | Text (e.g., “DNAEX12345”). |
| **Marker / Gene Region** | gene / amplificationTarget | Genetic region sequenced (typically COI-5P). | Text. |
| **Sequence ID / Accession** | geneticAccessionNumber / associatedSequences | Identifier of the sequence in BOLD or GenBank. | Text (e.g., “GBMK123456”). |
| **Sequence** | DNA_sequence / associatedSequences | DNA sequence associated with the specimen. | FASTA format. |
| **Sequence Length** | (no direct DwC equivalent) | Length of the DNA fragment in base pairs (bp). | Integer (e.g., 658 bp). |
| **Image(s)** | associatedMedia | Links to specimen photographs. | URL(s). |
| **Project Code / Project Name** | datasetID / datasetName | Code or name of the project within BOLD. | Text. |
| **Institution Storing DNA** | ownerInstitutionCode / preservationInstitution | Institution responsible for storing DNA or tissue samples. | Text. |
| **Voucher Type** | typeStatus | Type status of the specimen (voucher, paratype, holotype, etc.). | Text. |
| **Specimen Notes / Remarks** | occurrenceRemarks | Additional comments or notes about the specimen or collection. | Free text. |
| **Processing Status** | (no direct DwC equivalent) | Status of molecular processing (e.g., “sequenced”, “pending”, “failed”). | Text. |
| **BOLD Record URL** | references / associatedReferences | Direct link to the public record on the BOLD Systems portal. | URL. |

**Supplementary information 1. Outline of the twenty-five datasets at the BOLD repository**

The datasets of arthropod diversity in the Neotropical Eastern Pacific Bioregion are also available for direct download from the Barcode of Life Data Systems (BOLD) public data portal. These correspond to twenty-five datasets according to sampling localities listed along with their DOIs in Supplementary Table 1.

In BOLD, each dataset consists of specimens with a unique number identification number known as “Process ID”. Each individual specimen is associated with corresponding metadata described in the following subsections. Each dataset contains several data records. We first describe the dataset structure and its metadata, then the data record structure and its metadata.

Datasets: The datasets are hosted on the Barcode of Life Data Systems (BOLD) public data portal, each with a unique DOI number that serves as a hyperlink to access the dataset repository and metadata dashboard (Table S1). Each dataset is presented via a standardized, single-page web dashboard designed to provide an immediate summary of metadata, taxonomic coverage, geospatial distribution, and access to individual specimen records. The presentation layout is divided into five primary components:

1) Dataset Overview and Metadata. The dashboard header identifies the unique Dataset Code (e.g., DS-CRMCO3), providing a persistent identifier for citation. This section includes a Dataset Overview table that quantifies the record set's scope through the following metrics: specimens (total number of physical specimens collected), sequences (total count of genetic sequences obtained), records with BINs (number of records assigned a Barcode Index Number (BIN), a persistent registry for operational taxonomic units (OTUs), records with species (count of records identified to the species level), biodiversity metrics (total counts of unique BINs and Species represented in the dataset), geographic and institutional scope (a count of distinct countries/oceans and depository institutions involved).

2) Geospatial Visualization (Collection Sites). Geographic distribution is visualized through an interactive global map under the Collection Site(s) section. Sampling locations are plotted as data points, with a color-coded legend indicating specimen density per site (ranging from single records to clusters of 10,000+). This component provides an immediate visual assessment of the dataset's geographic range and sampling intensity.

3) Taxonomic and Temporal Visualizations. To illustrate the biological diversity and collection history of the dataset, the layout includes a Taxon Treemap with a hierarchical visualization that displays the relative abundance of records across taxonomic ranks. Another visualization is provided by section Collection Activity, which is a temporal histogram or chart depicting the frequency of specimen collection over time. The taxonomy component is integrated with external authoritative sources including the Catalogue of Life, ITIS, GBIF, WoRMS, and LepIndex. Within this taxonomic framework, each specimen DNA barcode sequence is assigned to a BIN by the Refined Single Linkage (RESL) algorithm28.

4) Specimen Records Table. The granular data are presented in a searchable and scrollable Records table at the bottom of the dashboard. This table enumerates individual specimen entries, displaying key attributes for each record. Based on the dataset's standard schema, the presented attributes typically include: Specimen ID / Sample ID (unique identifiers for the physical specimen), Taxonomy (identification at various levels, including BIN), Marker Code (the specific genetic marker sequenced), Location (country of collection), Institution (the holding institution for the specimen), and Collection Date (the date the sample was collected).

5) Data Access and Interoperability. To facilitate reuse and analysis, the interface provides direct download options in standard biodiversity data formats. Users can export the full dataset or subsets via the header links in JSON, TSV, or DwC formats. Additionally, the system links to specific APIs for programmatic access to summary statistics, images, and sequence data.

Data records: Individual records are presented via a standardized, single-page layout designed to aggregate all biological, geospatial, and molecular data associated with a single physical specimen. The layout consists of modular sections that provide granular detail, ensuring traceability and reproducibility. The primary components of the specimen record presentation include:

1) Header and Record Identification: The top of the page displays the Process ID (e.g., CRICA050-22), which is the unique, persistent identifier for the record within the BOLD architecture.

2) Specimen Imagery: A dedicated Specimen Images section displays high-resolution photographs of the physical voucher. These images are essential for morphological verification and are often accompanied by a Creative Commons license, ensuring open access for non-commercial use.

3) Geospatial Visualization: The Collection Site(s) component features an interactive map pinning the exact location where the specimen was collected. This visual aid allows for immediate verification of the geographic context and is color-coded to indicate sampling density if multiple records share the same coordinates.

4) Sequence Data and Traceability: The Sequence section provides the molecular data derived from the specimen. It includes: a) Sequence ID & Metadata: The Sequence ID & Metadata with links to the specific sequence entry (e.g., COI-5P) and the sequencing center. b) Nucleotide Sequence: The full DNA barcode sequence is displayed in text format, along with the sequence length (e.g., 653 bp). c) Primers: Information on the forward and reverse primers used for amplification. d) Trace Files: When available, links to the electropherogram trace files are provided for quality assessment.

5) Attribution and Ownership: To ensure proper credit and data governance, the Attribution section lists the institutions and individuals responsible for the data. This includes: 1) Specimen Depository: The physical repository holding the voucher specimen (e.g. Centre for Biodiversity Genomics). b) Collectors & Identifiers: The researchers who collected and taxonomically identified the specimen. c) Sequencing Center: The facility responsible for generating the genetic data. d) Trace Files, when available, links to the electropherogram trace files are provided for quality assessment.

6) Identifiers and Cross-Referencing: The Identifiers table acts as a cross-referencing hub, linking the BOLD record to other internal and external systems. Key attributes include: a) Sample ID / Museum ID: The identifier assigned by the holding institution. b) Field ID: The code assigned during fieldwork. c) BIN ID: A link to the Barcode Index Number (BIN) page, placing the specimen within a genetic cluster (OTU). d) Associated Datasets: Direct links to the parent dataset (e.g., DS-CRMCO3) or project (e.g. GMP - Cocos Edge Trap).

7) Other Metadata Sections: Specific metadata categories include: a) Taxonomy: A hierarchical breakdown of the specimen's classification, from Kingdom to Species/Subspecies, including the identification method and taxonomic notes. b) Specimen: Details on the physical voucher, including sex, life stage, tissue descriptor, and preservation status. c) Collection: Comprehensive fieldwork data, including the exact collection date, coordinates (latitude/longitude), elevation, habitat description, and sampling protocol (e.g., Malaise Trap). d) Ecology: Ecological context, assigning the record to a specific Biogeographical Realm, Biome, and Ecoregion (e.g., Neotropics, Tropical & Subtropical Moist Broadleaf Forest).
